# SPIRITUAL DEVELOPMENT AND AGING HUMANITIES: READING HESSE'S STEPPENWOLF THROUGH MOODY'S FIVE STAGES OF THE SOUL

**DOI:** 10.1093/geroni/igac059.405

**Published:** 2022-12-20

**Authors:** Stephen Fogle

**Affiliations:** University of Nebraska at Omaha, Omaha, Nebraska, United States

## Abstract

This paper explores Herman Hesse’s Steppenwolf through Harry Moody’s Five Stages of the Soul. Steppenwolf speaks to gerontological education objectives as it deals squarely with humanizing old age though, “deeply lived spiritual events which [the main character] has attempted to express by giving them the form of tangible experiences.” (Hesse, 1963, 21) Five Stages of the Soul is a seminal conceptual model for understanding spiritual development across the life course: particularly in old age. It documents lived experiences in five stages: call, search, struggle, breakthrough, and return. This paper follows Steppenwolf’s main character, Harry Haller, though each of these five stages. In doing so, this paper positions aging and spiritual development center stage in a tale featuring themes of enduring struggle such as personal identities, war & violence, sex, magic, and technological advancement. The plot line of Steppenwolf matches Five Stages of the Soul with enlightening consistency. Originally published a century ago, Steppenwolf enjoyed resurgent popularity among the baby boom cohort of older adults in the United States and globally during the 1960s. Today, Steppenwolf retains relevancy for understanding aging and spiritual development in lived contexts of social chaos and uncertainty. Steppenwolf is a classic and engaging piece of literature for contemporary readers of all ages. This paper helps students and teachers of gerontology apply a conceptual model of aging to a relevant case study. The paper contributes to incorporating literary masterpieces into gerontological curricula. Finally, this paper sheds light on the humanity of older adults engaged in spiritual development.

